# Benign elevations in serum aminotransferases and biomarkers of hepatotoxicity in healthy volunteers treated with cholestyramine

**DOI:** 10.1186/2050-6511-15-42

**Published:** 2014-08-03

**Authors:** Rohit Singhal, Alison H Harrill, Francoise Menguy-Vacheron, Zaid Jayyosi, Hadj Benzerdjeb, Paul B Watkins

**Affiliations:** 1 Sanofi, Disposition, Safety and Animal Research, Framingham, MA 01701, USA; 2 The Hamner-University of North Carolina Institute for Drug Safety Sciences, 6 Davis Drive, Research Triangle Park, NC 27709, USA; 3 Sanofi, 1 Ave Pierre Brossolette - 91385 Chilly-Mazarin Cedex, Paris, France; 4 Schools of Medicine, Pharmacy and Public Health, University of North Carolina at Chapel Hill, Chapel Hill, NC 27599, USA

**Keywords:** Liver, Biomarkers, Toxicity, Hepatotoxicity

## Abstract

**Background:**

There are currently no serum biomarkers capable of distinguishing elevations in serum alanine aminotransferase (ALT) that portend serious liver injury potential from benign elevations such as those occurring during cholestyramine treatment. The aim of the research was to test the hypothesis that newly proposed biomarkers of hepatotoxicity would not significantly rise in serum during elevations in serum ALT associated with cholestyramine treatment, which has never been associated with clinically relevant liver injury.

**Methods:**

In a double-blind placebo-controlled trial, cholestyramine (8g) was administered for 11 days to healthy adult volunteers. Serum from subjects with elevations in alanine aminotransferase (ALT) exceeding three-fold the upper limit of normal (ULN) were utilized for biomarker quantification.

**Results:**

In 11 of 67 subjects, cholestyramine treatment resulted in ALT elevation by >3x ULN (mean 6.9 fold; range 3–28 fold). In these 11 subjects, there was a 22.4-fold mean increase in serum levels of miR-122 relative to baseline, supporting a liver origin of the serum ALT. Significant elevations were noted in mean levels of necrosis biomarkers sorbitol dehydrogenase (8.1 fold), cytokeratin 18 (2.1 fold) and HMGB1 (1.7 fold). Caspase-cleaved cytokeratin 18, a biomarker of apoptosis was also significantly elevated (1.7 fold). A rise in glutamate dehydrogenase (7.3 fold) may support mitochondrial dysfunction.

**Conclusion:**

All toxicity biomarkers measured in this study were elevated along with ALT, confirming the liver origin and reflecting both hepatocyte necrosis and apoptosis. Since cholestyramine treatment has no clinical liver safety concerns, we conclude that interpretation of the biomarkers studied may not be straightforward in the context of assessing liver safety of new drugs.

## Background

Hepatotoxicity is a major reason for termination of clinical drug development programs and regulatory actions on drugs, including withdrawal from the market. The potential for a new drug candidate to cause clinically important liver injury is often difficult to ascertain in clinical trials due to deficiencies in the currently available serum tests, which have not changed in more than four decades. The most sensitive and widely employed serum test for liver injury is serum alanine aminotransferase (ALT). However, there are drugs that cause serum ALT elevations, even pronounced elevations, that do not cause clinically important liver injury [1]. To address this issue, the current FDA guidance for evaluation of drug-induced liver injury (DILI) in clinical trials recommends continuing treatment of subjects with asymptomatic ALT elevations (up to 8 × ULN) to determine whether there is subsequent loss of liver function, specifically with concurrent elevation of serum bilirubin (“Hy’s Law” criteria) [2]. However, this practice may be placing clinical trial participants at risk. There exists at least one example of a patient in a clinical trial who developed fatal liver failure despite discontinuing treatment with the implicated drug prior to the occurrence of elevation in serum bilirubin [3].

When ALT elevations are caused by drugs not known to cause clinically important liver injury, it has been assumed that the underlying mechanisms may not involve hepatocellular damage. Proposed mechanisms include extrahepatic sources of the ALT, leak of ALT from healthy hepatocytes, increased ALT synthesis within the liver, and/or reduced ALT clearance from blood. New serum biomarkers have been proposed to detect liver specific injury (miR-122), apoptosis (caspase cleaved cytokeratin-18 fragments), and necrosis (sorbitol dehydrogenase, full-length cytokeratin-18, glutamate dehydrogenase and HMGB1) [4-9]. It is a reasonable hypothesis that these new biomarkers may help distinguish between benign ALT elevations and those that portend potential for clinically important liver injury.

Cholestyramine, a cholesterol lowering drug, is an orally administered and non-absorbable ion-exchange resin that binds bile acids in the intestine, interrupting their enterohepatic circulation and increasing their fecal excretion [10]. Cholestyramine is also sometimes used to interrupt enterohepatic circulation of certain drugs and to accelerate their removal from the body [10,11]. To our knowledge, and in spite of the very large clinical experience with cholestyramine during decades of use, there have not been reports of clinically important hepatotoxicity associated with this treatment. Indeed, cholestyramine is commonly used in patients with advanced liver disease to relieve symptoms associated with cholestasis [12]. However, in at least two clinical studies cholestyramine treatment was reported to be associated with elevations in serum ALT [13,14].

In a clinical study involving treatment of healthy adult volunteers with cholestyramine, we observed asymptomatic elevations in serum ALT. This discrepancy between traditional interpretation of serum ALT and clinical safety experience raises the possibility that cholestyramine-associated elevations in serum ALT do not reflect true hepatocyte injury. To provide insight into mechanisms underlying these benign elevations of serum ALT, we assayed serum samples from subjects who experienced serum ALT elevations during treatment with cholestyramine for the liver specific microRNA, miR-122, and for biomarkers associated with necrosis and apoptosis. Our hypothesis was that these biomarkers would not demonstrate concomitant elevations with the serum ALT and may therefore be useful in clinical settings to distinguish benign elevations in serum ALT from those that portend potential for clinically important liver injury.

## Methods

### Study design

**Figure 1:**
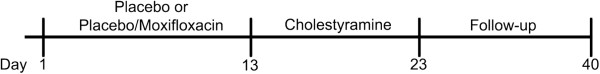
**The study design.** Subjects were administered placebo from day 1 to 12 of the study or placebo from day 1 to 12 and single dose 400 mg Moxifloxacin (Moxi) on day 12, followed by a washout phase with cholestyramine from day 13 to 23.

The subjects studied were a subset of a larger randomized, double blind, phase-I clinical trial conducted in healthy male and female subjects aged 18–65 years old in order to evaluate the potential for an active investigational new drug to prolong the QT interval. After a 12 day treatment phase (active drug or placebo), all subjects (including those randomized to placebo) entered a “washout” phase involving treatment with cholestyramine (8g powder, three times a day taken orally in a glass of fruit juice, milk soup or water, for 11 days, i.e., from day 13 to 23 of the clinical trial) or activated charcoal to accelerate the elimination of the investigational drug (in those that received active drug). The 67 subjects involved in the current study were all randomized to receive placebo during the treatment phase, never received the active drug, and only received cholestyramine during the washout phase. Thirty-four of these placebo treated subjects also received a single oral dose of moxifloxacin (positive control for QT prolongation), on the day before the start of cholestyramine treatment. The study protocol is shown in Figure 1. A complete description of the clinical trial has been included in CONSORT flow chart (Additional file 1) and CONSORT checklist (Additional file 2: Table S1).

Serial blood samples were obtained and serum enzymes (ALT, AST, and ALP) and total bilirubin were assayed before (baseline) and during the cholestyramine treatment period using AU 400 Olympus clinical chemistry analyzer at the Department de Biologie Clinique Centre régional de lutte contre le Cancer Eugene Marquis, Rennes Cedex, France. Samples were archived only from subjects who experienced confirmed ALT >3 times the ULN; only the baseline and the serum sample with the peak ALT value was archived from these subjects and available for biomarker analysis. The study protocol was reviewed and approved by the regional ethics committee, CPP OUEST VI, at the Centre Hospitalier Universitaire Cavale Blanche, Brest Cedex, France. The clinical trial was conducted in accordance with the International Conference of Harmonisation Guidelines for Good Clinical Practice and the Declaration of Helsinki. Written informed consent was obtained from all subjects prior to the study.

Only samples from subjects treated with cholestyramine after receiving placebo during the active treatment phase were analyzed in this study (i.e., no subjects studied received the investigational drug at any time).

### Investigational protein biomarker measurements

Analysis was performed on baseline serum samples and the single serum samples containing the highest value for ALT level (i.e., the only post-baseline serum sample archived and therefore available for analysis). Serum concentrations of glutamate dehydrogenase (GLDH; Randox Laboratories, Kearneysville, WV) and sorbitol dehydrogenase (SDH; Genzyme Diagnostics, Cambridge, MA) were determined using a commercial kit by quantifying the decrease in absorbance according to the manufacturer’s protocol. Assay results for both GLDH and SDH were quantified using the Cobas Fara II clinical chemistry analyzer (Roche Diagnostics, Indianapolis, IN). Caspase-cleaved and total K18 were determined using the M65 and M30 (apoptosense) ELISAs, respectively, in accordance with the manufacturer’s guidelines (Peviva, West Chester, OH). Total HMGB1 content was determined in serum by enzyme-linked immunosorbent assay (ELISA) per the manufacturer’s protocol (IBL International, Hamburg, Germany). For all ELISA analytes, the inter- and intra-assay variability was <10%.

### RNA isolation and miR-122 quantification

RNA isolation and miR-122 quantification were performed according to a protocol published previously [15], with the exception that 200μl serum was used for RNA extraction. The expression levels of miRNA hsa-miR-122 (miR-122) were quantified using the TaqMan miRNA reverse transcription kit and miRNA-specific stem-loop primers (Applied Biosystems, Foster City, CA) in a scaled-down (5μl) reverse-transcription reaction as described by Kroh *et al.*[16], using the 7900HT instrument (Applied Biosystems). MiRNA was quantified by absolute quantification via a standard curve, with quantities normalized to an exogenous spike-in control derived from *Caenorhabditis elegans*, cel-miR-39.

### Statistical analysis

Clinical biochemistry parameters were expressed as fold change from upper limit of normal (ULN). Biomarker values were expressed as post-dosing fold change compared to the pre-dosing baseline. Cholestyramine-mediated changes in the concentrations of biomarkers in comparison to the pre-dosing concentrations were assessed using paired one-tailed Student’s t-test. Biomarker correlation analyses were performed by calculating the Pearson correlation coefficient (*σ*). Descriptive statistics for liver-function tests and biomarkers included the arithmetic mean, standard error of the mean (SEM), and median. Differences in mean or correlation were deemed significant when *p* < 0.05.

## Results

### Clinical biochemistries

**Figure 2:**
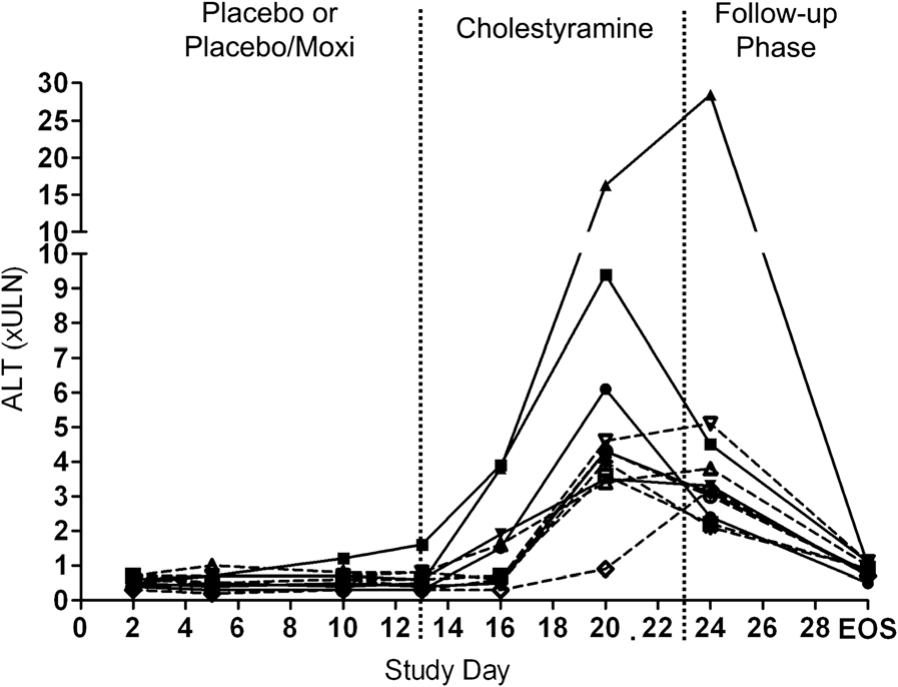
**Alanine aminotransferase (ALT) measurement.** Blood was collected before dosing on day 1 (baseline), and days 2, 5, 10, 13, 16, 20, 24, and at end of the follow-up phase. Solid and dashed lines represent placebo and moxifloxacin treatments, respectively. EOS is End of Study. Values represent fold change over upper limit of normal (ULN).

Of the 67 subjects who received placebo (and never received active study medication) and were administered cholestyramine, eleven subjects experienced asymptomatic elevations in serum ALT levels exceeding three-fold the upper limit of normal (>3× ULN). Six of these subjects had received the single oral dose of moxifloxacin the day before the start of cholestyramine treatment. The mean elevation was 255 U/L after treatment with cholestyramine compared to pre-dosing mean ALT level of 20 U/L. Four of the 11 subjects experienced peak ALT levels >5× ULN (2 men and 2 women) and one of the women experienced a 28-fold ULN increase (Figure 2). This latter subject was a 31 year old Caucasian woman with normal BMI (BMI = 25) taking oral contraceptives concomitantly. Two other young women with ALT elevation <5× ULN were also taking oral contraceptives. However, oral contraceptives were given to most of young women of child bearing potential in this trial and only one woman described above experienced an increase in ALT >5× ULN .

A rise in ALT levels was first evident three days after starting cholestyramine (study day 16), and the peak values were obtained 4 to 8 days later (between study day 20 and 24) (Figure 2). The single dose of moxifloxacin did not appear to influence the incidence or height of the serum ALT elevations observed; indeed, three subjects with the highest peak serum ALT values had not received moxifloxacin (Figure 2). The serum ALT rises were mirrored by elevations in serum AST (ρ = 0.99, *P* < 0.0001). The enzyme elevations were asymptomatic in each subject, returning to normal ranges within 2 weeks. There were no consistent changes in serum ALP or total bilirubin levels (Table 1) which indicated that there was no clinical evidence of cholestasis.

**Table 1:** Classical clinical chemistry parameters following cholestyramine administration in placebo- or moxifloxacin-treated subjects

**Subject#**	**Treatment type**	**ALT (U/L) baseline**	**ALT (U/L) peak**	**Peak ALT(ULN)**	**AST (ULN)**	**ALP (ULN)**	**Total bilirubin (ULN)**
1	Placebo	16	273	6.1	1.8	1.0	0.6
2	Placebo	19	320	9.4	3.6	0.7	0.4
3	Placebo	15	967	28.4	10.9	0.9	0.6
4	Placebo	18	119	3.5	1.9	0.7	0.5
5	Placebo	16	145	4.3	2.6	0.6	0.8
6	Placebo	12	146	4.3	2.6	0.6	0.4
7	Moxifloxacin	33	164	3.6	1.8	1.1	0.3
8	Moxifloxacin	32	173	3.8	2.0	0.6	0.5
9	Moxifloxacin	30	229	5.1	2.7	1.4	0.9
10	Moxifloxacin	14	142	3.2	1.5	0.7	0.9
11	Moxifloxacin	20	136	4.0	1.9	0.8	0.4

For ALT, both absolute peak value and ULN have been included. For AST, ALP, and total Bilirubin only ULN have been included.

### Experimental biomarkers

**Figure 3:**
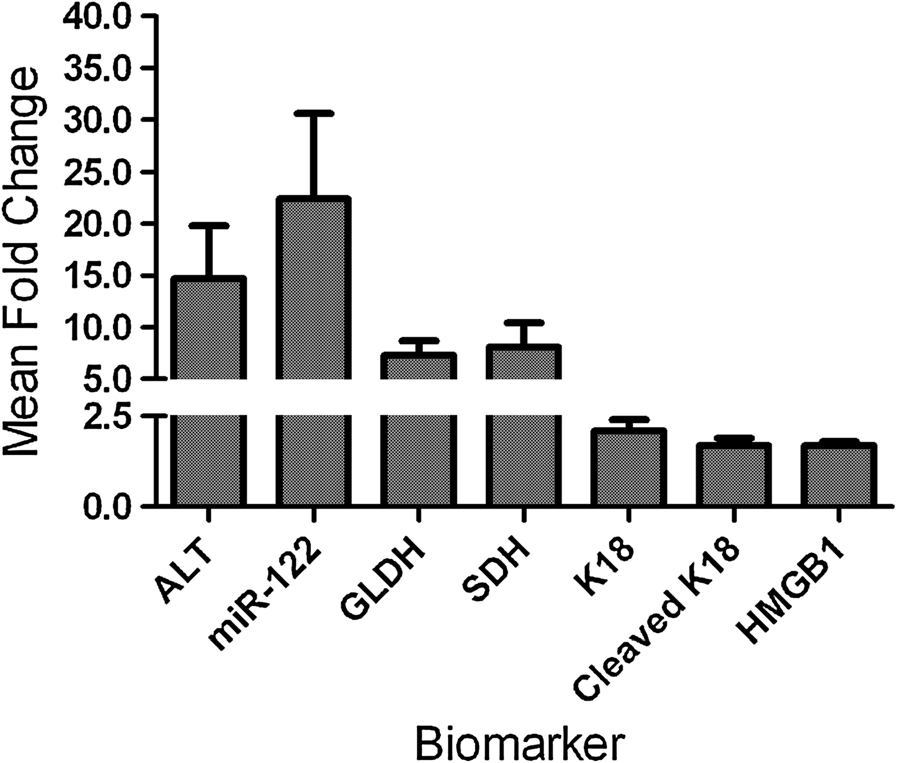
**Fold changes in investigational new biomarkers.** Values are mean ± SEM fold changes post vs pre cholestyramine administration, N = 11, represented as fold change post-dosing over pre-dosing.

Only the serum samples (baseline and peak) from subjects who experienced elevations in serum ALT exceeding 3 × ULN were archived and assayed for experimental biomarker analysis (Figure 3). The mean serum level of the liver-specific miRNA-122 [9] was elevated 22.4-fold over baseline (*P* < 0.05); mean levels of necrosis biomarkers SDH and HMGB1 [4,5,7,8,17] were elevated 8.1-, and 1.7 fold (*P* < 0.05), respectively over baseline. Mean serum levels of full-length cytokeratin 18 (M65) rose 2.1 fold, and its caspase-cleaved fragment (M30), a biomarker of apoptosis [5,6], also rose 1.7 fold (*P* < 0.05), Figure 3). Mean serum GLDH was elevated 7.3- fold. The single dose of moxifloxacin received by 5 of 11 subjects did not have any obvious influence on extent of elevation of any of the biomarkers measured relative to placebo. The Pearson correlation coefficient of peak ALT levels was 0.87 with SDH, 0.45 with GLDH, 0.53 with miR-122, 0.31 with cytokeratin 18, with a significant (*P* < 0.05) correlation observed between ALT and SDH, GLDH and SDH, and GLDH and full length K18 (Table 2). However, there was no correlation observed between fold elevation in serum ALT with fold elevation in caspase-cleaved K18 or HMGB1. Data obtained for each subject are listed in Additional file 3: Table S2.

**Table 2:** Pearson correlation between experimental new biomarkers and ALT

	**ALT**	**SDH**	**GLDH**	**miR-122**	**K18**	**Cleaved K18**	**HMGB1**
**ALT**		0.87*	0.45	0.53	0.31	-0.11	-0.16
**SDH**	0.87*		0.62*	0.39	0.44	0.22	0.08
**GLDH**	0.45	0.62*		0.11	0.83*	0.13	-0.25
**miR-122**	0.53	0.39	0.11		0.03	0.00	-0.52
**K18**	0.31	0.44	0.83*	0.03		0.29	-0.30
**Cleaved K18**	-0.11	0.22	0.13	0.00	0.29		0.17
**HMGB1**	-0.16	0.08	-0.25	-0.52	-0.30	0.17	

*Indicates significant correlation, P < 0.05.

## Discussion

Serum alanine aminotransferase (ALT) has for more than four decades been used in clinical trials as a sensitive means to monitor liver safety of new drug candidates. However, this biomarker lacks specificity and can be elevated during treatment with drugs that are entirely safe for the liver [1]. There is urgent need for new biomarkers capable of distinguishing ALT elevations observed in a clinical trial that represent a “false positive” – *i.e.,* the drug does not cause clinically significant hepatotoxicity vs. a “true positive” – *i.e.,* the drug has potential to cause progressive and clinically important liver injury. Cholestyramine has been used for the treatment of hypercholesterolemia for more than fifty years and this treatment has never been associated with clinically important liver injury. Hence, the marked elevations in serum ALT we observed during treatment with cholestyramine represent a “false positive” safety signal for this drug. In other words, if cholestyramine were a new drug in early clinical development, the profile of serum ALT elevations observed would likely result in termination of the development program over liver safety concerns although no true safety liability exists. Our serum sample set obtained from healthy adult volunteers treated with cholestyramine therefore provided a means for investigating false positive results using additional toxicity biomarkers that have been proposed to represent advances in multiple respects over traditional liver safety biomarkers.

The eleven healthy volunteers who experienced elevations in serum ALT during cholestyramine treatment also had significant increases in serum miR-122. Circulating miR-122 is reported to be essentially liver-specific and has been established as a marker of drug-induced liver injury in humans [9]. In fact, a recent rodent study suggested a greater specificity and sensitivity of peak serum miR-122 with the extent of liver necrosis as judged histologically than that of peak serum ALT [18]. The marked rise in miR-122 we observed confirms at least a substantial contribution of liver-specific injury to the biomarker elevations we observed during cholestyramine treatment. In addition, it is doubtful that miR-122 and the multiple liver proteins assayed share pathways for degradation or elimination in serum, indicating that accumulation in serum due to reduced clearance is unlikely.

Cholestyramine treatment produced increases in mean serum levels of GLDH, SDH, and HMGB1 which have been proposed as specific indicators of necrosis [8,17]. SDH is located primarily in liver and kidney; activity of SDH increases rapidly in acute episodes of liver necrosis [19]. HMGB1 is a non-histone nuclear protein that is abundant in the nucleus of cells [20]. The elevations we observed in the serum levels of these biomarkers support the occurrence of hepatocellular necrosis, rather than membrane blebbing or other mechanisms that could result in cytosol leakage from viable hepatocytes.

We also observed a rise in the serum concentration of caspase-cleaved cytokeratin18 fragments (M30) which supports occurrence of hepatocellular apoptosis due to cholestyramine administration [5,6]. Because it is possible for a toxic injury to result in apoptosis when sufficient cellular ATP levels are maintained, but progress to necrosis as the injury is more severe, we examined whether the ratio of cleaved to total cytokeratin 18 differed between those subjects with highest and lowest levels of ALT or miR-122 levels. No differences were evident, (Additional file 4: Table S3) so a shift from apoptosis to necrosis with injury progression was not supported by these data. The reasons why this low-level and clinically insignificant injury never progresses toward a more serious toxicity are not clear.

The mechanisms by which cholestyramine treatment could cause mild hepatocyte necrosis/apoptosis, and why this is without any clinical consequence was not the focus of our study and remains unclear. Cholestyramine is neither metabolized nor absorbed from the GI tract so it could not have a direct effect on the liver. We speculate that the interruption of enterohepatic cycling of essential fat-soluble molecules and/or bile acids by cholestyramine may lead to alterations in membrane integrity or compensatory increase in bile acid synthesis, and this may indirectly result in transient necrosis and activation of apoptotic pathways. However, cholestyramine administration leading to increased bile acid synthesis has not been associated with significant impacts on hepatocytes, which appear to adapt to the change [21]. There is experimental evidence that cholestyramine increases activation of c-JUN N-terminal protein kinase (JNK) in the context of acetaminophen overdose in mice, but this activation is not present without the acetaminophen challenge [22]. Interestingly, cholestyramine feeding alone did not alter hepatocellular levels of glutathione, indicating that cholestyramine itself does not elicit a pro-oxidant or injurious response, even at high exposure levels comprising 2% of the diet [22]. Bertolotti *et al.* observed that *in vivo* administration of cholestyramine induced a significant dose-related increase of 7α-hydroxylation along with a decrease in the concentrations of plasma cholesterol [23]. A decrease in the concentrations of circulating bile acids trigger a disinhibition of cholesterol 7α -hydroxylase, also known as CYP7A1, resulting in enhanced production of bile acids and reduced level of cholesterol in hepatocytes. Long term cholestyramine treatment also decreased the intestinal expression of genes involved in drug transport, cholesterol metabolism, and apoprotein synthesis - MRP2, ABCG5, ABCG8, SHP and SREBP-1c, and increased the hepatic expression - HMG-CoA reductase, CYP7A1, LDL receptor and LXRα genes [24,25]. How changes in the lipid, cholesterol and bile acid flux and altered hepatic metabolism by cholestyramine could rapidly translate into hepatocyte death is unclear but the rise in serum GLDH we observed may provide some insight. GLDH is a large protein localized in the mitochondrial matrix; its presence in blood reflects loss of mitochondrial integrity. Hence, it could be concluded that rises in serum GLDH during liver injury implicate mitochondrial dysfunction as a mechanisms underlying the necrosis [26]. Certain bile acids have been shown to cause mitochondrial dysfunction [27] and this could provide a mechanistic link between interruption in enterohepatic cycling, the proposed compensatory increase in bile acid synthesis, and hepatocyte death. One caveat is that our serum was not specifically prepared to eliminate mitochondria prior to freezing, and our serum samples underwent a freeze-thaw cycle which may have caused damage to intact mitochondria with leakage of detectable GLDH [26].

We have recently applied the same biomarkers used in the current study to serum samples obtained in healthy volunteers treated with various heparins [15]. Heparins, like cholestyramine, are known to cause marked elevations in serum ALT and AST, but these elevations are considered safe since heparins have not been reported to cause clinically important liver injury. In the heparin study, we also observed elevations in miR-122, serum GLDH and HMGB1 and concluded that mild and transient hepatocellular necrosis was occurring. However, despite the fact that the mean elevations of serum ALT and miR-122 are comparable in both the heparin and current cholestyramine studies, the serum HMGB1 level rose an average of 6.3-fold baseline in the heparin study but only a 1.7-fold increase was observed in the current study. Since we had only single serum samples from each subject, we could not test the hypothesis that this apparent discrepancy resulted from the kinetics of release and clearance of HMGB1. It should also be noted that in contrast to our current observations with cholestyramine, we did not find any rise in caspase-cleaved cytokeratin-18 (M-30) in the heparin study and concluded that heparins do not cause apoptosis. Because little HMGB1 is released from cells undergoing apoptosis [28], a reasonable conclusion is that the component of apoptosis caused by cholestyramine may in part explain the relatively low levels of HMGB1 we observed.

An important question is whether the magnitude of rise in the mechanistic biomarkers observed during benign ALT elevations (due to heparin or cholestyramine) is lower than would be observed during comparable ALT elevations produced by drugs capable of causing severe liver injury. If so, then there may be a threshold of concern that could be determined. Owing to the recent discovery and validation efforts for these biomarkers, standard ranges for human studies have not been established and, thus, these biomarkers may be more informative for determining tissue origin and gaining mechanistic insight. A recent publication by Antoine *et al.* investigated the same experimental liver injury biomarkers in the context of acute acetaminophen-induced liver injury in the clinic [29]. Comparison of the data from the acetaminophen study is complicated by the differences in dose, hospital presentation times, and study enrollment times of the acute injury patients. However, the values for serum ALT, GLDH, M65, and M30 measured in the present cholestyramine study were inclusive within the ranges observed for patients who developed clinically significant liver injury due to acetaminophen overdose (Additional file 5: Table S4). The observation could be partially explained by differences in sample collection and instrumentation between the two studies. However, this finding appears to support the assertion that the magnitude of elevation for GLDH, M65, and M30, does not strictly indicate a potential for overt liver injury in a given patient or study subject.

## Conclusion

Our biomarker data support the conclusion that the elevation in serum aminotransferases that occurs as a result of cholestyramine treatment reflects both necrosis and apoptosis of hepatocytes. This was an unexpected finding since cholestyramine has never been reported to cause clinically important liver injury. Caution must therefore be used when interpreting elevations in these markers in the context of assessing liver safety of new drug candidates in clinical trials. We believe that the application of these and other new biomarkers will have a future role in monitoring liver safety in clinical trials but additional validation studies with DILI negative and DILI positive compounds in both nonclinical and clinical settings are needed.

## Abbreviations

ALT: Alanine aminotransferase; AT: Aminotransferase; M65: Cytokeratin-18 full length; M30: Cytokeratin cleaved; DILI: Drug-induced liver injury; GLDH: Glutamate dehydrogenase; HMGB1: High-mobility group box- 1; MiR-122: MicroRNA-122; SDH: Sorbitol dehydrogenase; ULN: Upper limit of normal.

## Competing interests

Authors: A.H. had no support from any organization for the submitted work; no financial relationships with any organizations that might have an interest in the submitted work in the previous 3 years. R.S., F.M-V, Z.J., and H.B. are currently employed and have had support from Sanofi in the previous 3 years. P.W. serves as a scientific advisor to Sanofi. There are no other relationships or activities that could appear to have influenced the submitted work.

## Authors’ contributions

RS, AH, and PW analyzed the biomarker data. FM-V, ZJ, and HB designed the clinical protocol and helped analyze the biomarker data. All authors contributed to the writing of the manuscript.

## Pre-publication history

The pre-publication history for this paper can be accessed here:

http://www.biomedcentral.com/2050-6511/15/42/prepub

## Supplementary Material

Additional file 1 CONSORT 2010 Flow Diagram. Click here for file

Additional file 2 CONSORT 2010 checklists. Click here for file

Additional file 3 Pre and post-dosing values for investigational protein biomarkers. Click here for file

Additional file 4 Ratio of cleaved to total cytokeratin 18 in individuals with largest and smallest elevation in ALTs. Click here for file

Additional file 5 A comparison of Cholestyramine vs. Acetaminophen-induced elevation in biomarkers of liver safety. Click here for file
