# Supplementary material for: Benign elevations in serum aminotransferases and biomarkers of hepatotoxicity in healthy volunteers treated with cholestyramine
Source: BMC Pharmacol Toxicol. 2014 Aug 3;15:42. doi: 10.1186/2050-6511-15-42 (PMC4130124; doi:10.1186/2050-6511-15-42)
Supplement: Additional file 1 — CONSORT 2010 Flow Diagram. [file 2050-6511-15-42-S1.doc]

**
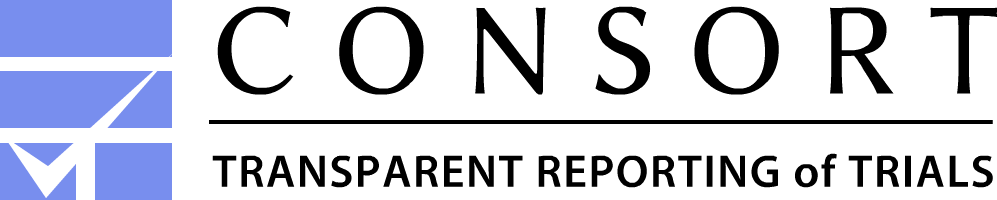
**

**CONSORT 2010 Flow Diagram**

**Allocation**

**Safety Analysis**

**Follow-Up**

**Enrollment**

Assessed for eligibility (n=195)

Excluded (n=1)

♦  Not meeting inclusion criteria (n= 0 )

♦  Declined to participate (n= 0 )

♦  Other reasons (n=1 )

Analysed (n=61 )
♦ Excluded from analysis (n=0)

Lost to follow-up (give reasons) (n=0)

Discontinued intervention (n=2 for adverse events )

Allocated to Active Drug (n= 61)

♦ Received allocated intervention (n=61 )

♦ Did not receive allocated intervention (n=0)

Allocated to cholestyramine post-treatment: n=31

Allocated to activated charcoal post treatment: n= 30

Lost to follow-up (give reasons) (n=0)

Discontinued intervention (give reasons) (n=0)

Allocated to placebo/ moxifloxacin (n=68)

♦ Received allocated intervention (n=68 )

♦ Did not receive allocated intervention (n=0)

**Allocated to cholestyramine post treatment: n= 34 ***

Allocated to activated charcoal post treatment: n= 34

Analysed (n=68 )
♦ Excluded from analysis (n=0 )

Randomized (n=194)

Allocated to placebo (n= 65)

♦ Received allocated intervention (n=61 )

♦ Did not receive allocated intervention (n=0)

**Allocated to cholestyramine post treatment: n = 33 ***

Allocated to activated charcoal post treatment: n= 32

Lost to follow-up (give reasons) (n=0)

Discontinued intervention (give reasons) (n=0 )

Analysed (n=65 )
♦ Excluded from analysis (n=0)

*those 67 subjects (33 placebo + 34 placebo/moxifloxacin) were the only subjects investigated in the current study for liver effect of cholestyramine
